# Microbicides Development Programme: design of a phase III trial to measure the efficacy of the vaginal microbicide PRO 2000/5 for HIV prevention

**DOI:** 10.1186/1745-6215-10-99

**Published:** 2009-10-27

**Authors:** Andrew Nunn, Sheena McCormack, Angela M Crook, Robert Pool, Clare Rutterford, Richard Hayes

**Affiliations:** 1 MRC Clinical Trials Unit, UK; 2 Barcelona Centre for International Health Research (CRESIB), Universitat de Barcelona, Barcelona, Spain; 3 Queen Mary University of London, Centre for Health Sciences, UK; 4 London School of Hygiene and Tropical Medicine, UK

## Abstract

**Background:**

With 2.5 million new HIV infections per year, effective preventive methods against HIV are urgently needed, especially in sub-Saharan Africa. MDP301 is an ongoing trial of the vaginal microbicide PRO 2000/5 being conducted by the Microbicides Development Programme. The main objective of the trial is to determine the efficacy and safety of 0.5% and 2% concentrations of PRO 2000/5 gel compared to placebo in preventing vaginally acquired HIV infection.

**Methods/Design:**

MDP301 is a multicentre randomised placebo-controlled Phase III trial. The design was informed by pre-trial feasibility and pilot studies. The choice of trial population, assessments and endpoints are discussed along with statistical and ethical considerations. Adaptations to the design were made during the conduct of the trial; these included closing a study arm and changing the timing of the primary endpoint.

**Discussion:**

The development of effective microbicide products remains one of the strongest hopes for new biomedical prevention tools. MDP301 is the largest Phase III microbicide trial to date, with 9404 enrolments, and is scheduled for completion in September 2009. Results are expected towards the end of 2009.

**Trial registration:**

Current controlled trials ISRCTN64716212.

## Background

The global pandemic of HIV infection continues to be one of the world's most pressing public health problems. After 25 years there is still little progress in reducing the spread of the epidemic which has had such widespread social and economic effects on poor communities in developing countries particularly in sub-Saharan Africa. With an estimated 2.5 million new infections in 2007[1], there is an urgent need for effective prevention measures.

In the face of such high prevalence and incidence, it is likely that a range of complementary interventions will be needed to bring HIV epidemics under more effective control. Thus, while behavioural modification and male circumcision [2-5] have an important role to play, other prevention measures need to be developed and tested. It is unlikely that an effective HIV vaccine will be available within the next ten years. The male condom represents the best available barrier method of protection, and is highly effective if used correctly and consistently. Unfortunately, regular condom use is particularly difficult to achieve among married couples or others in long-term relationships, and is clearly inappropriate for couples wishing to have children. An effective vaginal microbicide could offer the possibility of providing protection in such circumstances.

The results of the COL-1492 trial evaluating the effectiveness and safety of Nonoxynol-9 (N9), a licensed spermicide, in the prevention of vaginally acquired HIV infection in commercial sex workers, confirmed that development of a topically applied agent to prevent HIV was going to be challenging[6]. The increased risk of HIV in women using N9, and correlation between frequent use of N9 and genital ulceration, informed the need for a wide therapeutic index in vitro and absence of genital toxicity in Phase I/II trials when selecting future candidates. More recent microbicide trial results, although disappointing, have not, like N9, demonstrated an obvious harmful microbicide effect [7-10]. Although the Cellulose Sulphate trial was stopped prematurely for harm based on an interim result, the final reported results did not show significant evidence of harm[8].

At the time when the N9 results were released (June 2000), five research groups were planning programmes of research to evaluate six candidate microbicides. One of these candidates was PRO 2000/5, a naphthalene sulphonate polymer which disrupts the attachment and fusion steps in HIV infection of target cells. In this paper, we describe the rationale and design of an ongoing multi-centre Phase III trial of PRO 2000/5 which is being carried out by the Microbicides Development Programme. This is the largest Phase III microbicide trial to date, with 9404 enrolments, and is scheduled for completion in August 2009. We also discuss adaptations to the design that have been made during the conduct of the trial.

The main objective of the trial, designated MDP301, is to determine the efficacy and safety of 0.5% and 2% concentrations of PRO 2000/5 gel compared to placebo in preventing vaginally acquired HIV infection.

## Methods/Design

### Study populations

The trial is being carried out in six study locations in Eastern and Southern Africa, each coordinated by an academic institution or collaboration that is a partner in MDP. Each location has a single data management centre responsible for one or more clinic sites. Multiple research clinics were needed to provide an adequate sample size for the study in a timely manner, but also have the advantage of producing results which should be of greater generalisability.

**Figure 1:**
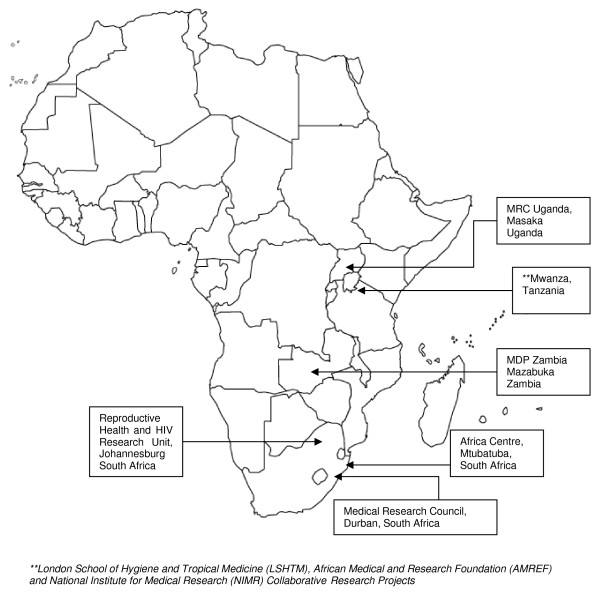
**Map of MDP301 clinical trial sites in Africa**.

The six study locations are shown in Figure 1 (map) and the populations are described in the Appendix. These populations were selected because they were known to be at moderate-to-high risk of HIV infection and because HIV incidence was expected to be sufficiently high to meet the sample size requirements of the trial. Prior to the trial, Feasibility Studies were carried out by each of the partners to confirm this and to inform sample size calculations. The three South African partners recruited HIV-uninfected women initially from health centres and family planning clinics but then through word of mouth and community hotspots in poor urban settlements close to Johannesburg and Durban, and from a rural population in KwaZulu Natal that forms part of a demographic surveillance system. These study populations are considered to be broadly representative of women at risk in the general population in these areas. The Zambian site recruited women who were provided with health care through their employment, or their partner's employment, on a sugar estate near the town of Mazabuka, as well as women in Mazabuka.

HIV prevalence at the remaining two sites in Tanzania and Uganda is known to be lower in the general population than in the four sites described above[1] and so women were recruited from two populations at increased risk. In Mwanza City, Tanzania, the trial recruited women who work in food and recreational facilities, including local food stalls, bars, guesthouses and brew shops. These occupational groups are known to be at high risk of HIV and other sexually transmitted infections (STIs) because some of the women supplement their incomes through occasional transactional sex. Finally, in Masaka, Uganda, the trial recruited women who are in discordant partnerships, that is their husband or regular male partner is known to be HIV-positive. In order to preserve the confidentiality of the male partners, a small proportion of sero-concordant negative couples were also included.

In COL-1492, and previous microbicide trials, the study populations targeted were commercial sex workers. Due to the concern that toxicity may have occurred because of a very high frequency of sexual acts and therefore excessive use of the microbicide product, it was decided to target women who were less sexually active.

Taking this into consideration the eligibility criteria for the trial were as follows.

### Inclusion

◆ Women aged 16 years and above at enrolment in Masaka and Mwanza, or aged 18 years and above at enrolment in the South African and Zambian sites

◆ Likely to be sexually active at entry and during follow-up

◆ Willing to undergo HIV testing at screening and approximately 12 weekly intervals, and additionally if required to determine HIV status

◆ HIV negative at screening according to the local HIV testing algorithm

◆ Willing to receive the HIV result before randomisation

◆ Willing to use study gel as instructed

◆ Willing to undergo regular speculum examinations and genital infection screens

◆ Willing to have regular urine pregnancy tests

◆ Willing to receive health education about condoms

◆ Willing and able to give informed consent

### Exclusion

◆ Unable or unwilling to provide a reliable method of contact for the field team

◆ Likely to move permanently out of the area within the next year

◆ Likely to have sex more than 14 times a week on a regular basis during the course of follow-up

◆ Using spermicides regularly

◆ Pregnant or within 6 weeks postpartum at enrolment

◆ Has grade 3 clinical or laboratory abnormalities which are considered by the clinician or the Trial Management Group to make enrolment inadvisable

◆ Requiring referral for assessment of a clinically suspicious cervical lesion

◆ Treatment to the cervix, or to the womb through the cervix, within 30 days of enrolment

◆ Known latex allergy

◆ Participating, or having participated within 30 days of enrolment, in a clinical trial of an unlicensed product, microbicide, barrier method or any other intervention likely to impact on the outcome of this trial

◆ Considered unlikely to be able to comply with the protocol

### Feasibility and pilot studies

Feasibility Studies were carried out at the trial sites before the trial commenced to measure HIV incidence, pregnancy and retention rates and to evaluate behavioural characteristics and condom use in women from the proposed study populations in a context of risk reduction counselling and condom promotion. Some of the key findings from the Feasibility Studies are summarised in Table 1. These data informed the sample size assumptions for MDP301, both in terms of the estimate of the HIV incidence in the control arm and retention of participants. Among 3174 women enrolled in these studies, there were 120 subsequent incident cases of HIV infection corresponding to incidence rates which ranged from 3.5 to 12.6 per 100 woman-years of observation across the sites. The weighted estimate of HIV incidence, adjusting for the proportion of participants each site was expected to be able to contribute to the trial, was 6.2 per 100 woman-years. We decided to use a conservative estimate of 4 per 100 women years for HIV incidence in the control arm as we thought it possible that women that correctly identified themselves at high risk may have been the first to come forward to participate in the Feasibility Studies, and that the introduction of gel in the trial may further facilitate condom use in all participants as well as the possibility that rates in the population might decline with time. Retention fell below 75% at three of the six locations by 40 weeks, and this supported a concern that adherence to gel and to scheduled clinic visits might decline with time. It was decided that the endpoint for the primary analysis should be at 40 weeks - but to monitor adherence to gel closely in the trial. At the request of the US Food and Drug Administration (FDA), all women were continued in the trial until 52 weeks to obtain longer term safety data.

**Table 1:** Feasibility Study data.

	**South Africa**			**Zambia**	**Tanzania**	**Uganda**
	Durban	Joburg	Africa Centre sites	Mazabuka	Mwanza^+^	Masaka*

Populations	Health clinics and associated communities				Recreational facilities	Sero-discordant couples

Start date	Aug 02	Oct 02	Jul 03	Mar03	Mar 03	Oct 03

N screened	1263	1088	882	1974	1573	1370

% HIV +ve at screen	47%	20%	50%	30%	25%	7%

% pregnant at screen	1%	4%	1%	4%	10%	-

N enrolled	608	757	453	590	1573	50

% of enrolled seen at FU or later†						

3 m	94%	84%	63%	87%	83%	90%

6 m	88%	79%	56%	79%	79%	84%

9 m	82%	83%	56%	70%	70%	86%

12 m	67%	87%	58%	63%	71%	86%

Recruitment period (months)	14	15	12	18	14	2

Person years FU	499.2	531.4	158.1	356.4	717.4	31.2

Sero-conversions	37	21	20	13	25	4

HIV incidence	7.4	3.9	12.6	3.6	3.5	12.6

95%CI	5.4, 10.2	2.6, 6.1	8.2, 19.6	2.1, 6.3	2.4, 5.2	4.8, 34.1

†follow-up rates based on % of 3 month attendees who returned subsequently; +All 1573 women were enrolled in Mwanza but the HIV incidence calculation was based on those who were HIV negative at enrolment;*sero-discordant couples enrolled

Following the Feasibility Studies, a Pilot Study was initiated to assess the trial case record forms (CRF), procedures for pharmacy and informed consent, the database and the acceptability of the placebo gel. In particular, a procedure for verifying the accuracy of answers to questions on adherence and sexual practices in the CRF was assessed. This involved cross-checking reported product use against returned applicators and triangulating self-reported gel use and sexual behaviour data from the CRF with data from coital diaries and in-depth interviews. Ethical and regulatory approval for the Pilot Study was obtained in all sites. The Pilot Study was completed in all sites before commencing enrolment into the trial. At each stage of the protocol development there was continuous consultation with trial sites before the protocol, CRFs and trial procedures were finalised.

### Study arms

The MDP301 trial was designed to compare two alternative concentrations of the PRO 2000/5 gel, 0.5% and 2%, to a matching placebo. The randomisation was 1:1:1 and the sample size was calculated to provide adequate power for the comparison of each of the active gel arms with the placebo arm. The use of a single placebo arm to evaluate the efficacy of two alternative products is a more efficient design than carrying out two separate trials. The rationale for choosing a low and a high dose of PRO 2000/5 was that whilst the highest tolerable dose was likely to provide the greatest biological efficacy against HIV, the possibility that minor, undetectable, local toxicity could increase the risk of HIV infection had to be considered.

The possibility of incorporating an additional condom-only control arm for which no gel is provided was given consideration, with the rationale that the placebo gel may provide some protection against infection, diluting any treatment effect.

The inclusion of a condom-only arm was not included for three main reasons. First, the placebo being used was thoroughly tested and found to have negligible activity against HIV *in vitro*, and to afford no protection in a mouse model for vaginal HSV-2 infection. Second, women in the no gel arm would not be blind to their treatment allocation. This could result in a change in their sexual behaviour and condom use, with unpredictable effects on HIV incidence and any treatment effects. While reported condom use may be recorded, such data are subject to reporting and recall bias, which may be differential between study arms, and it is unlikely that estimated treatment effects could be corrected adequately for behavioural differences. Third, women in the condom-only control arm may be less motivated to complete the trial and, if there is higher loss to follow-up in this arm, this would introduce bias resulting in distortion of efficacy estimates.

For these reasons, only a placebo control group was included in the MDP301 trial. This choice was made recognising that some dilution of effect due to protection from the placebo gel could not be ruled out. However, providing the active gel cannot be identified by participants, requiring for example that there are no distinguishing adverse or other effects, adequate blinding of study arms ensures that risk behaviour will be comparable and that any observed difference in HIV incidence can be confidently ascribed to the active gel.

### Study schedule

**Figure 2:**
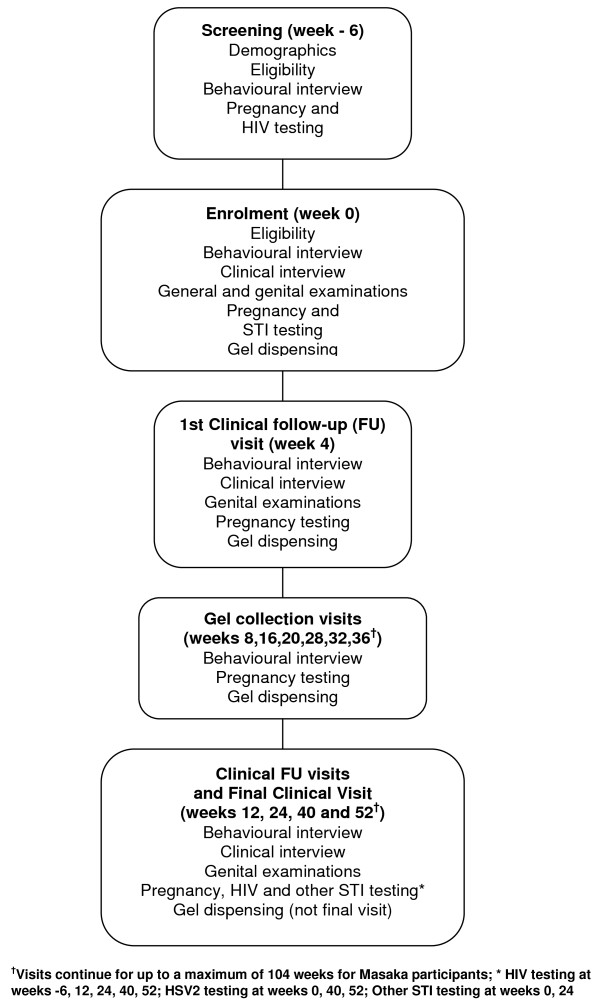
**Summary of visit schedule for MDP301 participants**.

A summary of the study schedule of visits and procedures is shown in Figure 2. Women attended an initial screening visit at which eligibility for the trial was assessed. Only women who were known to be HIV-negative were enrolled in the trial, and so the screening visit included HIV testing and counselling. Women were asked to return for the randomisation visit up to 6 weeks later and, if they satisfied all the eligibility criteria and gave informed consent, they were enrolled in the trial.

From the date of enrolment, women are being followed up for 52 weeks and are seen every 4 weeks for provision of gel. At each of these visits, sufficient gel is dispensed to carry them through to the next visit, with a buffer stock in case of late or missed visits. Women are asked to bring used and unused gel applicators with them to each monthly visit, and careful counts are made to ensure full gel accountability and to allow gel exposure estimates.

At enrolment and at the 4, 12, 24, 40 and 52 week visits, additional procedures are carried out according to the schedule. These include a clinical evaluation with genital examination, and the collection of specimens for the diagnosis of HIV, incident herpes simplex type 2 infection (HSV2), syphilis, *Neisseria gonorrhoea (NG)*, *Chlamydia trachomatis (CT), Trichomonas vaginalis (TV)*, bacterial vaginosis (BV) and candida.

At every follow-up visit, a urine specimen is tested for pregnancy and all pregnant women are told to interrupt gel use, but are asked to remain under follow-up. The protocol was amended to allow women to restart gel use following a negative pregnancy test, normal pelvic examination and 3 to 6 weeks after termination/early miscarriage or delivery respectively. Data are also collected on outcome of pregnancy.

Routine laboratory parameters for haematology, coagulation tests and biochemistry were collected from the first 500 women enrolled at the Durban and Johannesburg research clinics, and also (following a request from the FDA to include a non South African site) all the women enrolled in Masaka.

At weeks 4, 24, 40 and 52 women are interviewed in more detail about sexual behaviour and vaginal practices.

In the Masaka clinics only, women are followed for between 52 and 104 weeks, depending on when they were randomised, to provide longer-term information on gel safety.

### Randomisation and blinding

A randomisation list for each clinic was created using a computerised random number generator by an independent statistician at the MRC Clinical Trials Unit (CTU) in London, under the supervision of the Trial Statistician, AJN. Each list contained unique trial numbers matched to a series of 9 drug product codes, as manufacture of trial product took place in several batches. The list matching drug product codes to each of the three trial arms was kept separately and was available only to these two statisticians, the additional independent statistician who assists with unblinded data for the Independent Data Monitoring Committee and those responsible for packaging and labelling at the manufacturing plant.

Each eligible woman providing informed consent was given a unique trial number selected sequentially from the trial register held in each clinic. The pharmacist uses this trial number and drug product codes to dispense the appropriate allocation using the randomisation list. The dispensed boxes of gel applicators are labelled with the trial number identifier.

The link between trial number and drug product codes is available to the three statisticians referred to above, pharmacy personnel and the database designer at CTU. No personnel at the study sites have any access to the randomisation list, and there is no documentation at the sites that permits unblinding of product allocation. A hard copy of the list and a password protected database linking drug product codes to trial numbers is available only to pharmacy staff.

These procedures ensure that randomisation concealment is achieved and that the study is fully blinded, including participants, staff at the study sites, staff at CTU monitoring trial data, and laboratory personnel.

### Endpoints

#### Primary endpoints

The primary efficacy endpoint is the incidence of HIV infection in participants confirmed to be HIV negative at enrolment. HIV serological testing is carried out on sera collected at enrolment and at the 12, 24, 40 and 52 week clinic visits (and later visits in Masaka), using site-specific HIV testing algorithms. All suspected HIV seroconversions are further evaluated by a central reference laboratory using an algorithm that incorporates DNA PCR performed on cells from the buffy coat collected at week 0.

One of the unique aspects of HIV prevention trials is that the primary efficacy endpoint is also a primary safety endpoint because the possibility of increased risk of acquiring HIV-infection as a consequence of using the gel cannot be ruled out. In addition to HIV incidence, all deaths and severe or life-threatening clinical and laboratory events confirmed on examination or repeat testing will be primary safety endpoints.

#### Secondary endpoints

Pre-clinical data suggest that PRO 2000/5 also has activity against HSV-2, NG and CT. There are no suitable models for evaluation of activity against TV or BV. In light of the tests available for these pathogens and their natural history, it was decided to use sero-incidence of HSV-2, and cross sectional prevalence of NG and CT, to assess the efficacy of PRO 2000/5 in preventing these secondary endpoints.

Secondary safety endpoints are all systematically solicited genital adverse events (non-menstrual bleeding, epithelial disruption, erythema with and without discomfort, oedema, discomfort), and all clinical and laboratory adverse events.

### Behavioural measurements

Interpretation of the findings of the trial will depend on having reliable data on various aspects of behaviour. First, documenting the demographic and behavioural characteristics of the participants is necessary to evaluate the likely generalisability of the results to women in other populations. Second, data on usage of gel and condoms is essential in interpreting the measured effectiveness of the product. The term *efficacy *is generally used to denote the effect of an intervention under perfect conditions, for example implying 100% adherence and consistently correct use. In practice, such conditions cannot be achieved and microbicide trials will actually measure product *effectiveness*, this term denoting the effect under actual conditions of use. The level of effectiveness experienced in trials is, however, likely to be greater than that achieved in routine conditions.

During the trial, women are counselled to use condoms for all sex acts, but it is recognised that not all women will achieve consistent condom use. Since correct condom use is highly protective against HIV infection (and other STIs), it is important to monitor gel use during sex acts *unprotected *by condoms and make every effort to optimise this during the trial.

Intravaginal practices, such as vaginal cleansing, are known to be highly prevalent in some of the study populations and these may interfere with any protective effects of the gel. Although women are counselled not to engage in these practices at all, and especially not within one hour prior to applying the gel and for at least one hour after sex has occurred, it is recognised that they may not always comply with this guidance. Thus, measurement of these practices is also important.

Three main approaches are being used during the trial to record these variables. First, used and unused applicator returns are recorded and checked against the dispensing records. Second, structured questionnaires are used at each clinic visit to record details of use of gel and condoms at the last sex act, and at weeks 4, 24, 40 and 52 a longer questionnaire is used to capture data on additional sex acts, anal sex and intra-vagingal hygiene practices. Third, a random sample of at least 100 women at each site is asked to fill in pictorial diaries recording number of vaginal or anal sex acts, gel and condom use during individual sex acts, and intra-vaginal practices prior to the visits at weeks 4, 24 and 52. They are also interviewed in-depth on these same topics within days of the clinic visit and the results from all these sources are compared and triangulated. Details of these methods are provided elsewhere[11].

### Ethical considerations

The MDP301 trial is being carried out in accordance with international good clinical practice (ICH GCP) guidelines. Ethical permission to carry out the trial was given by local university or national research ethics committees in South Africa, Tanzania, Uganda and Zambia. Two ethics committees in the UK reviewed the protocol. The protocol was also reviewed by the national regulatory authorities in each participating country, as well as the Food and Drug Administration in the US where gel was manufactured. The trial commenced in October 2005 and completed enrolment in August 2008. Four versions of the protocol were implemented during this period on receipt of the necessary approvals.

### Informed consent

Informed consent was obtained through a two-stage procedure. Women were initially requested to give consent for screening and then subsequently, if they were eligible, for enrolment and randomisation. An interval of one to six weeks between the screening and enrolment visits allowed sufficient time for women to consider carefully whether they wished to take part. On both occasions, information was given to women about microbicides, the risks and benefits of taking part, confidentiality and their right to withdraw at any time, as well as details of the trial procedures. Information was provided during one-to-one interviews with the help of written materials and visual aids, and women were given the opportunity to raise any questions or concerns. Volunteers were next asked three questions that could not be answered yes or no to ascertain that they understood (a) that gel might not protect them from HIV, (b) that condoms do prevent HIV and (c) that they would have to stop using the gel if they became pregnant. Following this procedure, if women agreed to take part they were asked to indicate their consent by signature or thumbprint. If the woman was illiterate, an impartial witness of her choice was present to witness the discussion and her thumbprint consent.

Key points addressed during the informed consent procedure at enrolment were: (a) that the trial is being conducted to find out whether the gels will prevent HIV infection; (b) that there is a one in three chance that participants will receive the placebo gel and that no-one, including the staff, will know which product she is using; (c) that women are asked to use the gel during all sex acts and, because it is not known if the gels are active, that it is best if condoms are also used whenever possible; (d) that women will have to be withdrawn from gel if they become pregnant; (e) that symptoms such as genital irritation, unexpected bleeding, sores and ulcers should be reported to the study team regardless of whether the participant thinks they are related to product use; and (f) that they are asked to answer questions, especially those regarding gel use and sexual practices, as accurately as possible.

At specific visits during follow-up each participant's comprehension of the following is reviewed: a) that no-one knows whether any of the study products will protect against HIV or STI infections and so it is best to use condoms with gel; b) that they should contact the study staff before their next appointment if they have a problem such as bleeding or suspect that they might be pregnant; c) that they need to try to answer the questions about gel use accurately; d) that they are free to withdraw or stop using gel at any time.

### Standard of care

At the 12, 24, 40 and 52 week clinic visits, women are provided with HIV testing and counselling with promotion of safer sex practices, provision of free condoms, and diagnosis and treatment of sexually transmitted diseases. Women are advised to use a reliable method of contraception during the trial and several are available through the research clinics. A pregnancy test is carried out at every scheduled visit to the clinic. Women who become pregnant are withdrawn from study gel but asked to continue to attend their clinical follow-up visits, and will be followed up to record the outcome of pregnancy and included in a secondary MITT analysis (see below).

Women found to be HIV-positive at screening or who become HIV seropositive during follow-up are referred to local services for HIV treatment and care according to procedures developed at each study site.

### Trial oversight

MDP301 has an Independent Data Monitoring Committee (IDMC) which meets every 4-6 months to review the data on adverse events and HIV incidence by study arm and consider any emerging current literature. The IDMC is responsible for advising the Trial Steering Committee (TSC) of any changes that it recommends in the conduct of the study that may be required during the course of the trial. Because of the unique nature of the trial whereby HIV-seroconversion is the primary endpoint for both efficacy and safety the IDMC have had access to unblinded data on seroconversion rates by study arm at all of their meetings. No formal stopping rules were suggested for safety concerns. There was one planned efficacy interim analyses which took place after accumulation of half the expected woman-years, and the IDMC were to advise the TSC to stop the trial early in the event of overwhelming evidence of efficacy (p < 0.001). Adopting this approach allows for (approximate) preservation of the type I error rate at the end of the trial[12,13].

Community mechanisms have been established in each study site to facilitate a two-way flow of information between the study team and study population and to provide a forum where any concerns about the trial can be discussed.

### Sample size

At study onset, the proposed primary endpoint was HIV sero-conversion by the week 40 visit in a participant confirmed to be negative at enrolment. The enrolment target was originally 9673 women, to enable 2055 woman-years per arm to be accumulated by 40 weeks, allowing for 15% loss of woman-years due to withdrawals for pregnancy and loss to follow-up.

As indicated previously we assumed a conservative estimate of HIV incidence in the placebo arm of 4.0 per 100 woman-years. It was considered unrealistic to expect high efficacy against HIV incidence, and 40% was considered to be the highest efficacy likely to be achieved. Thus the power of the study to detect a 40% reduction in incidence in either of the active gel arms, compared with the placebo arm, as significant at the 5% level, would be 82%, increasing to 89% if incidence is 5.0 per 100 woman-years. The study was not powered for a comparison of the two active gel arms.

Following two protocol amendments described later, the primary endpoint was moved to week 52 and the sample size adjusted in order to achieve 80% power at the lower effect size of 35% reduction in HIV incidence. A revised estimate of 2640 woman-years per group is now required in order to achieve this.

Sample size calculations were also carried out for secondary and safety endpoints. Data from feasibility and other studies in the study sites indicated HSV-2 prevalence ranging from 50% to 85%, and HSV-2 incidence of approximately 10 per 100 woman-years. Assuming that 75% of the study population is already infected at enrolment and that HSV-2 incidence is 10 per woman-years then given approximately 1920 evaluable woman-years of observation at 52 weeks, the trial would have 90% power to detect a 50% reduction in HSV-2 incidence or 71% power for a 40% reduction. It is also proposed to compare the prevalence of NG and CT infection at the 24 week clinic visit. Assuming that approximately 7500 women are seen at this visit, there will be 89% power to detect a reduction in prevalence of either infection from 6% to 4%, 94% for a reduction from 3% to 1.5% or 59% power for a reduction from 3% to 2%. Assuming a minimum of 3000 women are enrolled in each study arm, there will be considerable power to detect small increases in rare adverse events, for example 97% power to detect an increase from 1.5% to 3%, or 80% power to detect an increase from 0.5% to 1.2% or 0.1% to 0.5%.

### Modified intention to treat analysis

The primary analysis will be a modified intention to treat (MITT) analysis in which women will be censored if they are withdrawn from gel because they become pregnant, but not if they are withdrawn for other reasons unless they refuse further follow-up. Following, the protocol amendment to allow women to restart gel following a negative pregnancy test, the additional woman-years accrued after re-starting gel will also be included in the MITT analysis.

Intention to treat analysis is usually recommended to preserve the comparability of study arms that is ensured by randomisation and to avoid selective exclusion of data on participants who may respond differently to the intervention from those who remain under follow-up throughout. The justifications for adopting a MITT approach in this trial are that (a) withdrawal for pregnancy is required by the study protocol for safety reasons and does not reflect a participant decision to withdraw which might be related to experience of the gel; (b) pregnancy rates are not expected to be influenced by gel use, so that the comparability of study arms ensured by randomisation should be maintained; and (c) while women are encouraged to use effective methods of contraception, pregnancy rates in all study sites remain substantial so that a classical intention to treat analysis would be expected to considerably under-estimate product efficacy and thus to reduce the power of the study.

### Closure of 2% study arm

Following a review of the data accrued by 15^th ^January 2008, the IDMC recommended that the 2% gel arm should be terminated on the grounds of futility, indicating that continuation of this study arm would be unlikely to demonstrate significant benefit of this concentration of PRO 2000/5. This recommendation was accepted by the TSC, and women remaining on 2% gel at that time were withdrawn from gel, but asked to continue to attend the long clinic visits at weeks 4, 12, 24, 40 and 52.

The closure of the 2% arm provided an opportunity to redirect resources to maximise the precision and power of the trial for the 0.5% gel *vs *placebo comparison. It was decided to continue with the original enrolment targets, but with women randomised in a 1:1 ratio to the 0.5% and placebo arms.

**Figure 3:**
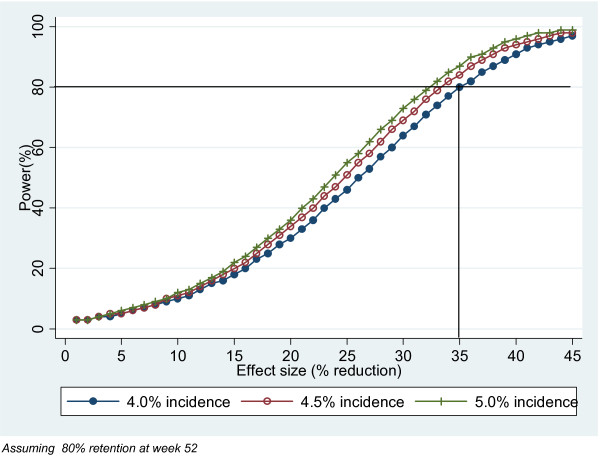
**Effect on study power when follow-up for primary endpoint increased from 40 to 52 weeks for different assumptions concerning adherence and HIV incidence**.

Figure 3 shows the estimated power of the study following the above design changes, with a 52 week primary endpoint and increased enrolment to the 0.5% and placebo arms. It shows that the trial will now have 80% power to detect a lower effect size of 35% assuming a control incidence of 4% (conservative) in the control arm. This increases to 85% and 88% assuming a control incidence of 4.5% and 5% respectively.

### Timing of primary endpoint

The primary endpoint was initially planned to be HIV incidence up to 40 weeks follow-up. It was argued that follow-up and adherence to gel use were likely to decrease over time and that a long duration of follow-up might therefore compromise the ability of the trial to demonstrate product efficacy. Given the need for proof of concept that HIV incidence can be reduced by a topical microbicide, a short follow-up period of 6 months or even 3 months might be adopted to maximise adherence and thus estimated product efficacy. However, this has to be weighed against the cost and complexity of enrolling very large numbers of women in order to accrue sufficient woman-years of observation with such a short follow-up period. The choice of 40 weeks follow-up was a compromise designed to ensure adequate adherence and follow-up without an undue increase in cost and logistical difficulty.

During the course of the trial, after monitoring adherence and follow-up rates in May 2008, it was clear that the initial concerns justifying the adoption of a shorter follow-up were not supported by the data. There was little evidence of fall off in follow-up between weeks 40 and 52, and all study sites had achieved follow-up of over 80% of expected participants at the 52 week visit. Moreover, adherence to gel had remained high with gel use reported at more than 85% of sex acts throughout follow-up. Therefore a change of time point for the primary analysis was considered due to the advantages of longer term follow-up and increased power with the increased woman- years.

Table 2 illustrates the trade-off between increased woman-years and potentially reduced adherence (implying a smaller treatment effect) for a primary endpoint of 52 weeks instead of 40 weeks, assuming HIV incidence in the placebo arm is 4.0 per 100 woman-years, with efficacy estimates ranging from 30% to 40% with 80% gel adherence up to 40 weeks. If gel adherence remains at 80% from 40 to 52 weeks (scenario 1), study power is increased substantially with a 52 week endpoint, from 85% to 90% for 40% effect size. Even if adherence falls as low as 50% from week 40 to week 52 (scenario 2), there is still a slight increase in study power with the 52 week endpoint (85% to 86%), and only a small change in the measured efficacy (37% compared to 40%). In the unlikely worst case scenario of the adherence dropping to 0% (scenario 3) then the corresponding power drops to 79% (from 85%) and efficacy measure to 34% (from 40%).

**Table 2:** Trade-off between increased woman-years (power) and falling adherence.

**Scenario**	**Week**	**Adherence**	**Observed reduction** in HIV incidence*	**95% CI**	**% power**
1. Adherence is unchanged from Week 40 to week 52	40	80%	40%	(17%, 56%)	85%
	52	80%	40%	(20%, 55%)	90%
	40	80%	35%	(11%, 52%)	72%
	52	80%	35%	(14%, 51%)	80%
	40	80%	30%	(6%, 48%)	55%
	52	80%	30%	(8%, 47%)	64%

2. Adherence drops to 50% at week 52	40	80%	40%	(17%, 56%)	85%
	52	50%	37%	(16%, 53%)	86%
	40	80%	35%	(11%, 53%)	72%
	52	50%	33%	(12%, 49%)	75%
	40	80%	30%	(6%, 48%)	55%
	52	50%	29%	(8%, 46%)	66%

3. Adherence drops to 0% at week 52	40	80%	40%	(17%, 56%)	85%
	52	0%	34%	(14%, 50%)	79%
	40	80%	35%	(11%, 52%)	72%
	52	0%	29%	(6%, 45%)	63%
	40	80%	30%	(6%,48%)	55%
	52	0%	25%	(2%, 42%)	50%

*Compared to placebo

All figures are based on following assumptions:4% HIV incidence in control arm; 85% retention at week 40; 80% retention at week 52

Based on these observations, a proposal was made to change the primary endpoint to HIV incidence by the week 52 visit, and this decision was endorsed by the TSC. This decision was made without disclosure of the accruing data on HIV incidence in the remaining two study arms.

## Discussion

In the era of roll-out of antiretroviral therapy, prevention of HIV infection has become an even more important priority since it will be very difficult to sustain adequate treatment services for those who need it unless the number of new infections can be brought under control. While there have recently been a number of disappointing results from trials of vaginal microbicides, the development of effective microbicide products remains one of the strongest hopes for new biomedical prevention tools.

Microbicide trials are large, complex and costly and it is important that they are designed and conducted carefully so as to maximise the precision of the results. Adequately powered trials ensure that the efficacy of an effective product can be established. Equally, if the product is ineffective, narrow confidence intervals ensure that such trials are able to rule out clinically important effects.

A number of special considerations in the design of this trial have been highlighted in this paper. First, the incorporation of two active gel arms, with different concentrations of PRO 2000/5, allows the comparison of each concentration with placebo more efficiently than in two independent two-arm studies. However, we decided against the incorporation of a fourth condom-only control arm, arguing that observed differences in HIV incidence between this arm and the gel arms would be very difficult to interpret. This could lead to greater confusion at the same time as increasing the cost and complexity of the trial.

Second, we chose to adopt for our primary analysis a modified intention to treat approach in which observations on women who become pregnant are censored at the time they are withdrawn from gel. While a full intention to treat analysis will also be carried out as a secondary analysis, we have provided a justification for the modified approach to be used for our primary analysis.

Third, when there is a pressing need to establish an effective microbicide as a new biomedical tool, it may be advisable to limit the follow-up period so as to maximise product adherence and minimise losses to follow-up. This helps to ensure that efficacy is not under-estimated. However, cost and logistical factors also need to be considered and a follow-up period that is very short would not be feasible since it would require the screening and enrolment of extremely large numbers of women. This is of particular concern in study populations with high HIV prevalence, since this increases the number of women who have to be screened to enrol the target number of HIV-negative women into the trial. We initially adopted a 40 week (9 month) primary endpoint for HIV incidence as a compromise between ensuring high adherence while minimising cost and sample size. As the trial proceeded, however, we changed to a 52 week (12 month) primary endpoint after observing that adherence and follow-up rates remained high beyond 40 weeks. No operational changes were necessary to implement this into the trial as all women were already being followed to week 52 for safety as requested by the FDA.

Fourth, measurement of behaviours such as frequency of sex acts and use of gel and condoms is very important in trials of this kind so that efficacy can be estimated from measured product efficacy, particularly in unprotected sex acts where condoms are not used. Triangulation of the behavioural data from a variety of quantitative and qualitative methods in this trial, including the use of coital diaries and in-depth interviews, will provide a measure of how reliable these data are, as well as an understanding of the factors influencing product use.

Finally, we have taken the opportunity provided by the premature closure of the 2% gel arm in this trial to redistribute resources so that precision of our findings on the 0.5% gel is optimised. Based on the observed overall HIV incidence during the trial, we will have high power of detecting an effect of clinical significance if this gel is effective. Increased power at this lower effect size has become even more relevant in light of the recent results from the Phase IIb study of 0.5% PRO 2000/5 gel which observed a 30% reduction in HIV incidence in the PRO 2000/5 gel arm compared to placebo, p = 0.1[14]. Enrolment to the MDP301 trial was completed in August 2008, follow-up will be completed in September 2009 and the results should be available by the end of 2009.

## Competing interests

The authors declare that they have no competing interests.

## Authors' contributions

AN and RH prepared the initial draft of the manuscript. SM, AC RP and CR all contributed with substantive revisions to subsequent drafts and all the authors read and approved the final manuscript.

## Appendix

### Description of study populations

1. 6805 women from communities with access to primary health care facilities in Durban (n = 2391), Johannesburg (n = 2500) and the Africa Centre sites (n = 1177), South Africa, and in Mazabuka, Zambia (n = 737)

2. 598 women who were entitled to primary care either through their employment or their partner's employment on the Nakambala sugar estate in Mazabuka, Zambia

3. 1146 women working in bars, hotels, guesthouses and other food or recreational facilities in or near Mwanza City, northern Tanzania

4. 840 HIV sero-discordant couples (and some sero-concordant negative couples to maintain blinding of sero-status) recruited in the Masaka district of Uganda from either office based voluntary counselling and testing services or following census and sero-survey
